# The diurnal pattern and social context of screen behaviours in adolescents: a cross-sectional analysis of the Millennium Cohort Study

**DOI:** 10.1186/s12889-022-13552-8

**Published:** 2022-06-07

**Authors:** Elli Kontostoli, Andy P. Jones, Andrew J. Atkin

**Affiliations:** 1 School of Health Sciences, Faculty of Medicine and Health Sciences, University of East Anglia, Norwich Research Park, Norwich, NR4 7TJ UK; 2 Norwich Medical School, Faculty of Medicine and Health Sciences, University of East Anglia, Norwich Research Park, Norwich, NR4 7TJ UK; 3 Norwich Epidemiology Centre, University of East Anglia, Norwich, NR4 7TJ UK

**Keywords:** Screen behaviours, Adolescents, Diurnal pattern, Social context, Time-use diary, Cross-sectional

## Abstract

**Background:**

Screen behaviours are highly prevalent in young people and excessive screen use may pose a risk to physical and mental health. Understanding the timing and social settings in which young people accumulate screen time may help to inform the design of interventions to limit screen use. This study aimed to describe diurnal patterns in adolescents’ screen-based behaviours and examine the association of social context with these behaviours on weekdays and weekend days.

**Methods:**

Time use diary data are from the sixth wave (2015/2016) of the Millennium Cohort Study, conducted when participants were aged 14 years. Outcome variables were electronic games/Apps, TV-viewing, phone calls and emails/texts, visiting social networking sites and internet browsing. Social context was categorised as alone only, parents only, friends only, siblings only, parents and siblings only. Multilevel multivariable logistic regression was used to examine the association between social contexts and screen activities.

**Results:**

Time spent in TV-viewing was greatest in the evening with a peak of 20 min in every hour between 20:00 and 22:00 in both sexes on weekdays/weekend days. Time spent using electronic games/Apps for boys and social network sites for girls was greatest in the afternoon/evening on weekdays and early afternoon/late evening on weekend days. Screen activities were mainly undertaken alone, except for TV-viewing. Compared to being alone, being with family members was associated with (Odds Ratio (95% Confidence Interval)) more time in TV-viewing in both boys and girls throughout the week (Weekdays: Boys, 2.84 (2.59, 3.11); Girls, 2.25 (2.09, 2.43); Weekend days: Boys, 4.40 (4.16, 4.67); Girls, 5.02 (4.77, 5.27)). Being with friends was associated with more time using electronic games on weekend days in both sexes (Boys, 3.31 (3.12, 3.51); Girls, 3.13 (2.67, 3.67)).

**Conclusions:**

Reductions in screen behaviours may be targeted throughout the day but should be sensitive to differing context. Family members, friends, and adolescent themselves may be important target groups in behaviour change interventions. Future research to address the complex interplay between social context, content and quality of screen behaviours will aid the design of behaviour change interventions.

**Supplementary Information:**

The online version contains supplementary material available at 10.1186/s12889-022-13552-8.

## Background

Screen behaviours are highly prevalent in young people and excessive screen use may contribute to an increased risk of cardio-metabolic syndrome, mental health disorders, and poor academic attainment [1–4]. The most prevalent screen activities include TV-viewing, tablet and smart-phone use [5], with data showing that more than half of young people exceed current screen-time recommendations of 2 h a day [6]. Considering that these behaviours track into adulthood [7], it is important for interventions to target them early in life.

Changing health behaviours requires an understanding of the factors that influence behaviour and the context in which they occur. The socio-ecological framework serves as a useful model for outlining the factors that might impact engagement in screen behaviours. This is because socio-demographic, environmental, and social factors play a key role in determining the accumulation of individuals’ screen time [8–10]. It is likely that humans behave differently in different contexts due to their innate ability to transform and connect in different ways at different times with a changing environment [11].

Several recent studies have examined the social context in which young people’s screen behaviour occurs, highlighting possible locations for the delivery of behavior change interventions [12, 13]. For example, previous research has shown that adolescents who spent more time alone after school reported higher screen-time than those who were with family or friends [13]. Much of this previous work, however, has focused on composite measures of screen time, aggregating data on different types of behaviour, such as TV-viewing and computer use. The Royal College of Paediatrics and Child Health advise against the use of composite screen-time markers in light of emerging evidence that the different behaviours may be differentially associated with health and wellbeing [14]. To mitigate health risks, the development of interventions therefore should be informed by understanding of the context in which specific screen-based activities take place.

In addition to understanding the social and environmental context of screen-based activity, understanding its distribution across the day may also be informative for intervention design, highlighting periods of the day when specific behaviours are likely to occur. Previous research has shown that accelerometer measured time spent sedentary was greater after-school than before or during school [15], with around half of this time spent using screens [12]. Evidence also suggests that the afternoon and evening period during weekends represents the largest accumulation of sedentary time [15]. However, our understanding is limited by the paucity of evidence regarding the timing of different types of screen activities throughout the day. There is evidence that sedentary behaviour patterns differ between boys and girls and that the determinants of these behaviours may also differ by sex [8], but we have limited information about how contextual factors may vary by sex. A recent study reported no difference by sex in where adolescents spent their after-school and weekday evening periods, or who they spent time with, but screen time was derived as a composite measure rather than by specific activity in that work, potentially masking true variation [13].

There is a need to better understand the timing and contexts in which screen behaviours take place if interventions to address them are to be targeted precisely. This evidence will help to identify which agents of change to target (i.e. parents, peers), where interventions should be implemented (e.g. home, school) and/or the time of day (e.g. preschool, evening) that intervention strategies should be activated [16]. The aim of this study, therefore, is to describe diurnal patterns in adolescents’ screen-based behaviours and examine the association of social context with these behaviours at weekdays and weekend days.

## Methods

### Sample and data collection

Data are from the Millennium Cohort Study (MCS), a national longitudinal birth cohort study run by the Centre for Longitudinal Studies at the University College London. The MCS examines the social, economic, and health related circumstances of young people born in 2000–2002, recruited from all four countries of the UK (England, Scotland, Wales and Northern Ireland) [17, 18]. The MCS was nationally representative at inception and 18,552 families (18 818 children) were recruited at baseline. Data collection has taken place when participants were 9 months, and 3, 5, 7, 11, 14, and 17 years of age. This cross-sectional analysis uses data from the sixth wave of assessment (MCS6; data collection: January 2015-April 2016), when participants were aged 14 years. In MCS6, 15,415 families were contacted for participation; 11,884 participants from 11,726 families provided partial or complete data. Parents and cohort members provided written and verbal consent prior to completing the survey [19]. The MCS6 was approved by the National Research Ethics Service, Research Ethics Committee London – Central (REC ref: 13/LO/1786). Data were anonymised and obtained from the UK Data Service (http://doi.org/10.5255/UKDA-SN-8156-7).

### Time-use diary

Participants were invited to complete a time-use diary, available in 3 formats: online via the web, App via tablet or phone, and paper. Sixty-four percent of participants selected the App diary format, 29% used the online version and 7% the paper diary. Participants completed the diary for two randomly chosen days (one weekday and one weekend day) with behaviour recorded in 10-min slots from 4 to 4am the next day. For each slot, participants indicated their main activity, selecting from a pre-specified list of 44 activities, nested within 12 categories (the full list of activity codes is presented in Additional file 1). In addition to reporting their main activity, cohort members also reported who they were with at that time, selecting from one or more of the following five options: alone, parents, siblings, friends, other adults.

Six screen-based activities were chosen for this analysis: electronic games and Apps, TV-viewing, phone calls, emails/texts, visiting social networking sites and internet browsing. Data were aggregated to mean minutes per hour spent in each activity, separately for weekdays and weekend days.

Reports of adolescents’ social context (i.e., ‘who they were with’) were coded into six categories: alone only, parents only, friends only, siblings only, parents and siblings only and other grouping (i.e., a combination of parents and friends and/or parents, friends and other adults).

### Covariates

Participants sex, family income, ethnicity, body mass index (BMI) and home location (rural or urban classification) were included as potential covariates in the analysis [20]. Indicators for home location were derived by geographically linked data across the four countries that specified whether participants were located in rural/urban areas based on population density [21]. Family income was measured using the Organisation for Economic Co-operation and Development (OECD) equivalised income quintiles, based on parent-reported household income. Ethnicity was parent-reported and categorised as White, Mixed, Indian, Pakistani and Bangladeshi, Black or Black British, and Other Ethnic group (including Chinese). Weight and height were measured by trained research assistants. Body mass index (BMI) was calculated as weight divided by height squared (kg/m^2^) and International Obesity Task Force (IOTF) thresholds were used to categorise participants as underweight/normal weight, overweight and obese [22].

### Data analysis

Analyses were conducted using STATA 16.0 (Stata Corporation, Texas, USA), with survey commands used to account for the stratified clustered design of MCS. Due to differences in the social and environmental contexts in which participants were immersed, analyses were conducted separately for week and weekend days. To describe diurnal patterns in each of the selected behaviours, data were aggregated to summarise duration (minutes) in each behaviour for each hour of the 24 h period of assessment. Social context information is presented as the proportion of time reported in each of the 6 contexts, separately for each behaviour of interest. Screen behaviour duration data were highly skewed; therefore, behavioural outcomes were dichotomised (no screen activity vs. screen activity) in the analysis of associations with social context. In addition, due to infrequent reports in phone calls, text/emails, using social network sites and internet browsing we created two composite outcomes for use in this analysis: (1) phones, texts, and emails, (2) using social network sites and internet browsing. Reports of TV-viewing and electronic games/apps were analysed individually. Multilevel multivariable logistic regression was used to assess associations between social contexts (i.e., who the adolescents were with) and screen activities. All models were adjusted for weight status, ethnicity, family income and home location. In preliminary analyses, we examined whether associations between social context and screen behaviours were moderated by sex, sibling status, ethnicity, socioeconomic position and family structure. Interaction terms were non-significant in all instances except for sex. Accordingly, all analyses were conducted separately for boys and girls. To account for the limited occurrence of screen-activities before and during school hours, weekday analysis of social context were restricted to the after-school period (15.00–23.00). Analyses of weekend data focussed on the full 24 h period.

## Results

**Fig. 1:**
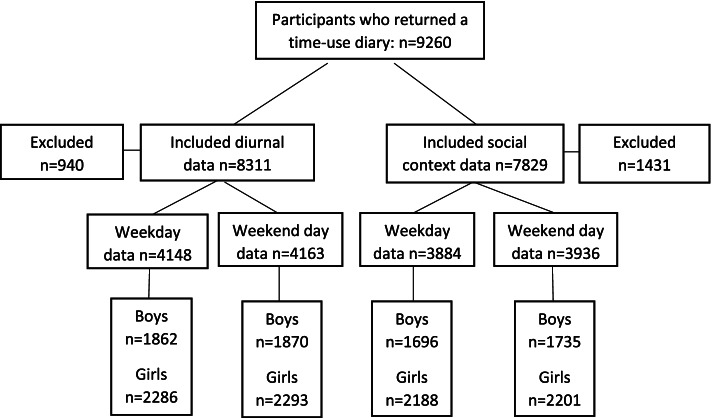
Participants who provided diurnal and social context data

Data were available for 9,251 diaries, of which 1,431 were excluded due to missing data on social context and 940 were excluded due to missing data on diurnal pattern. Figure 1 shows diary and data inclusion. The analytical samples for diurnal and social context analyses were *n* = 8,311 and *n* = 7,829 respectively. Drop-out analysis indicated that participants included in the analyses were more likely to be of white ethnicity (*P* < 0.001), have normal weight (*P* < 0.05) and come from families with higher income (*P* < 0.05) compared to those who were excluded.

### Diurnal patterns in screen activities

**Fig. 2:**
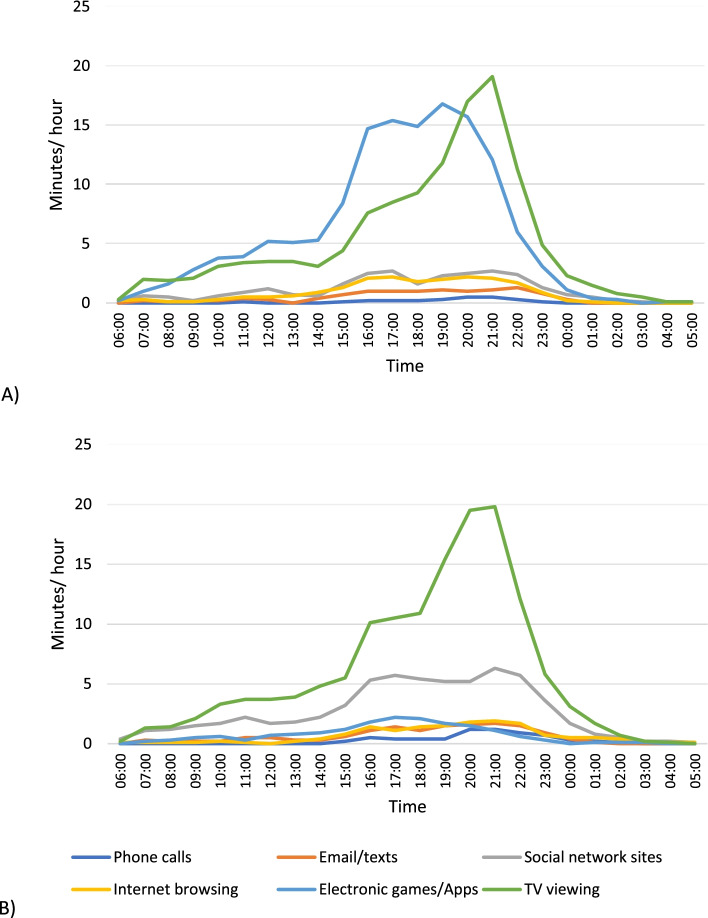
Minutes per hour spent in screen behaviours on weekdays: (**A**) boys, (**B**) girls

Figure 2 shows time spent in screen activities on a weekday, separately for boys and girls. Between midnight and 06:00, all screen behaviours accounted for less than 5 min in every hour. The most prevalent screen behaviour was TV viewing in both sexes, followed by electronic games/apps in boys and using social networking sites in girls. The time spent viewing TV was greatest in the evening, rising gradually from approximately 15:00 onwards to a peak of just under 20 min per hour between 21:00 and 22:00 for both sexes. In boys, the time spent using electronic games/Apps was greatest in the late afternoon and evening hours, rising from approximately 14:00 onwards to a peak of 15–17 min per hour between 16:00 and 19:00. The time spent using social network sites ranged of 5–7 min for girls. Time spent on the phone, sending emails / texts and browsing the internet peaked between the hours of 16:00 and 22:00, but remained low at approximately 2 min per hour for both sexes.

**Fig. 3:**
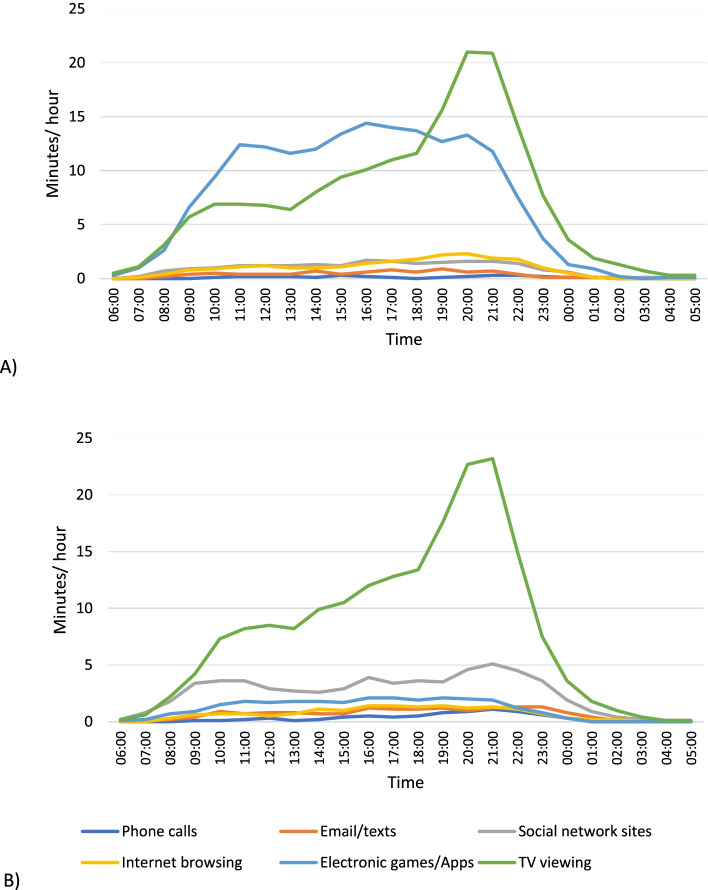
Minutes per hour spent in screen behaviours on weekend days: (**A**) boys, (**B**) girls

Figure 3 shows time spent in screen activities on a weekend day, separately for boys and girls. Between midnight and 06:00 all screen behaviours accounted for less than 1 min in every hour. The most prevalent screen behaviour was TV viewing in both sexes, followed by electronic games/apps in boys and using social networking sites in girls. The time spent viewing TV was greatest in the evening, but rose gradually from approximately 08:00 onwards, peaking at approximately 23 min between 20:00 and 21:00 for both sexes. In boys, use of electronic games/Apps was common throughout most of the waking day, averaging 10–15 min per hour between 11:00 and 21:00. In girls, use of social network sites was spread throughout the day accounting for 4–5 min per hour from 09:00–23:00. In both sexes, time spent on the phone, sending email/texts and browsing the internet remained low at approximately 2 min per hour throughout the day.

### Social contexts in screen behaviours

**Fig. 4:**
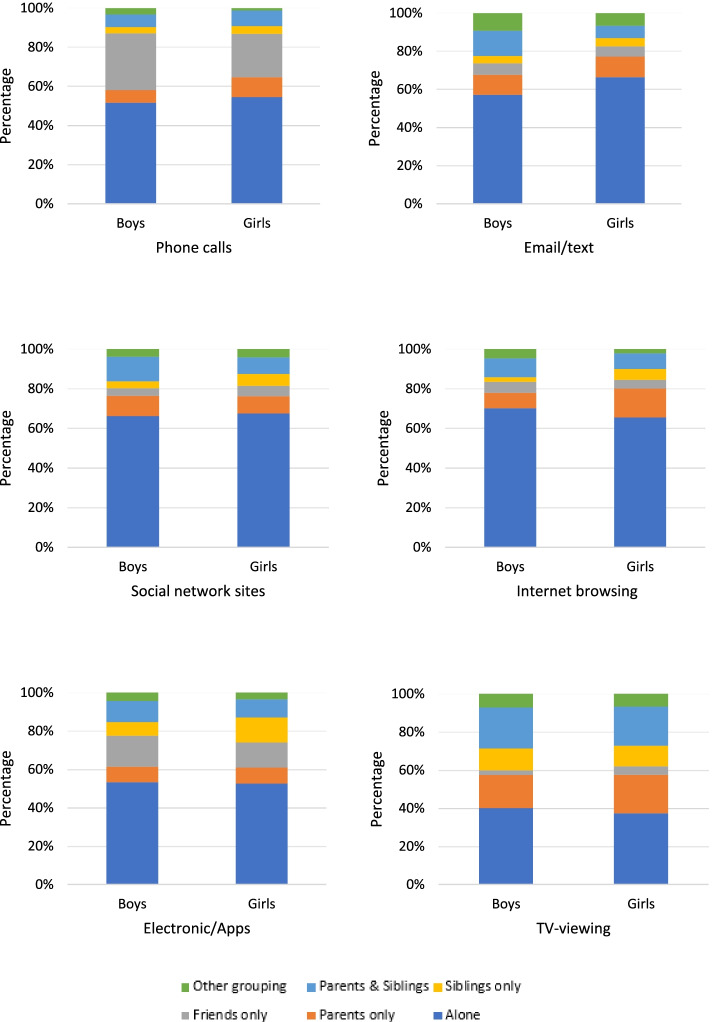
Social context of screen behaviours on a weekday, stratified by sex

**Fig. 5:**
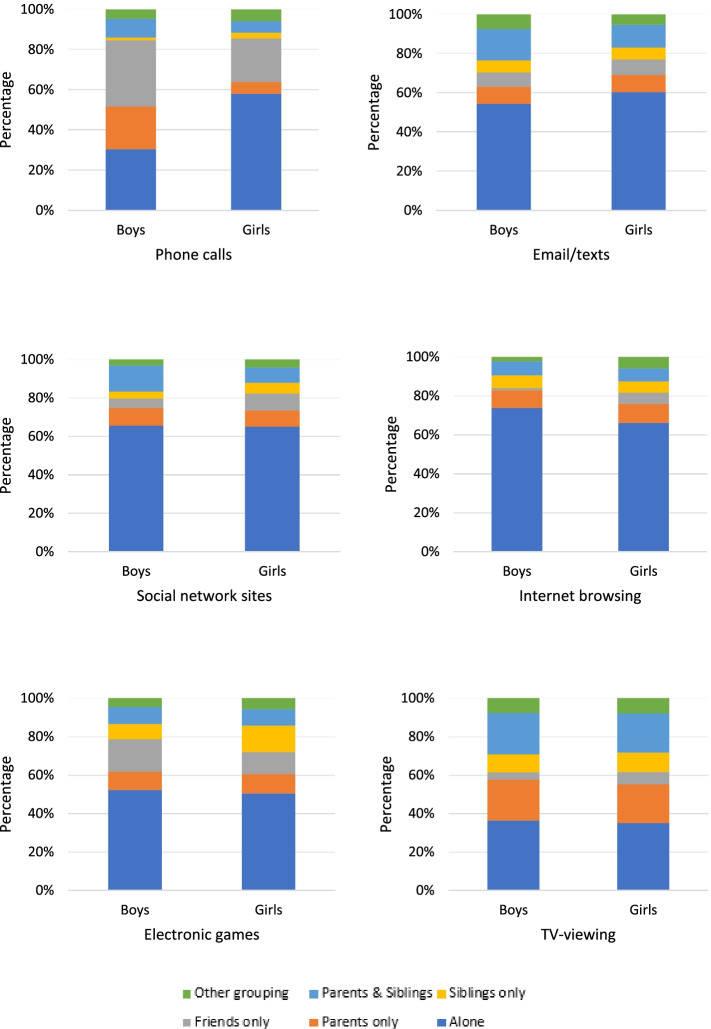
Social context of screen behaviours on a weekend day, stratified by sex

Figures 4 and 5 show social context of screen behaviours stratified by sex on a weekday and weekend day respectively. All the behaviours considered were undertaken alone for more than 50% of the time, except for TV viewing and phone calls at the weekend (boys only). Secondary to being alone, the most frequently reported contexts were ‘friends’ and ‘parents’, but these accounted for less than 20% of time spent in each behaviour. Approximately 40% of the time spent in TV-viewing, was undertaken alone, 20% of the time with parents only and 20% with parents and siblings. The only categories of behaviour frequently undertaken with friends were playing electronic games or making phone calls; this was the case on both week and weekend days.

### Associations between social contexts and screen behaviours on weekdays and weekend days

Associations between social contexts and screen-based activities on weekdays and weekend days stratified by sex are presented in Tables 1 and 2. Compared to the reference category of being alone, all social contexts were associated with lower odds of undertaking any of the behaviours studied on weekdays, with differences being highly statistically significant. In girls only, being with siblings was associated with higher odds of playing electronic games compared to being alone. Being with parents or siblings only and parents & siblings combined was associated with higher odds of time spent in TV viewing on a weekday in boys and girls.

**Table 1:** Cross-sectional association between social contexts and screen behaviours on a weekday in boys (*n* = 1805) and girls (*n* = 2180)

**Phone calls and Email/texts**				
	Boys		Girls	
	OR (95% CI)	*P* value	OR (95% CI)	*P* value
Alone	Reference group		Reference group	
Parents only	0.43 (0.21, 0.84)	0.01	0.28 (0.16, 0.47)	< 0.001
Friends only	0.34 (0.15, 0.78)	0.01	0.30 (0.17, 0.51)	< 0.001
Siblings only	0.52 (0.36, 0.74)	< 0.001	0.59 (0.22, 1.06)	0.08
Parents & siblings	0.25 (0.14, 0.34)	< 0.001	0.30 (0.25, 0.37)	< 0.001
Other grouping	0.23 (0.08, 0.65)	0.005	0.08 (0.03, 0.21)	< 0.001
**Social network and Internet browsing**				
	Boys		Girls	
	OR (95% CI)	*P* value	OR (95% CI)	*P* value
Alone	Reference group		Reference group	
Parents only	0.22 (0.13, 0.37)	< 0.001	0.22 (0.16, 0.32)	< 0.001
Friends only	0.04 (0.02, 0.09)	< 0.001	0.05 (0.03, 0.09)	< 0.001
Siblings only	0.28 (0.13, 0.58)	0.001	0.40 (0.26, 0.61)	< 0.001
Parents & siblings	0.22 (0.14, 0.35)	< 0.001	0.19 (0.14, 0.27)	< 0.001
Other grouping	0.08 (0.06, 0.10)	< 0.001	0.09 (0.05, 0.16)	< 0.001
**Electronic games**				
	Boys		Girls	
	OR (95% CI)	*P* value	OR (95% CI)	*P* value
Alone	Reference group		Reference group	
Parents only	0.21 (0.15, 0.32)	< 0.001	0.30 (0.13, 0.70)	0.006
Friends only	0.67 (0.46, 0.96)	0.03	0.56 (0.43, 0.74)	< 0.001
Siblings only	0.80 (0.70, 0.91)	< 0.001	2.03 (1.58, 2.60)	< 0.001
Parents & siblings	0.23 (0.16, 0.33)	< 0.001	0.46 (0.36, 0.59)	< 0.001
Other grouping	0.15 (0.13, 0.18)	< 0.001	0.14 (0.09, 0.21)	< 0.001
**TV-viewing**				
	Boys		Girls	
	OR (95% CI)	*P* value	OR (95% CI)	*P* value
Alone	Reference group		Reference group	
Parents only	2.28 (1.66, 3.13)	< 0.001	2.57 (2.11, 3.14)	< 0.001
Friends only	0.06 (0.03, 0.12)	< 0.001	0.12 (0.09, 0.17)	< 0.001
Siblings only	3.62 (2.47, 5.32)	< 0.001	3.00 (2.34, 3.86)	< 0.001
Parents & siblings	2.85 (2.15, 3.80)	< 0.001	2.48 (2.06, 2.98)	< 0.001
Other grouping	0.78 (0.69, 0.89)	< 0.001	0.64 (0.50, 0.83)	0.001

*OR* Odd Ratio, *95% CI* 95% Confidence Interval

**Table 2:** Cross-sectional association between social contexts and screen behaviours on a weekend day in boys (*n* = 1805) and girls (*n* = 2180)

**Phone calls and Email/texts**				
	Boys		Girls	
	OR (95% CI)	*P* value	OR (95% CI)	*P* value
Alone	Reference group		Reference group	
Parents only	0.80 (0.65, 0.97)	0.02	0.53 (0.36, 0.77)	< 0.001
Friends only	1.85 (1.59, 2.15)	< 0.001	0.93 (0.60, 1.42)	0.74
Siblings only	1.02 (0.48, 2.16)	0.94	0.60 (0.37, 0.98)	0.04
Parents & siblings	0.88 (0.46, 1.67)	0.70	0.52 (0.33, 0.81)	0.004
Other grouping	0.72 (0.55, 0.95)	0.02	0.37 (0.22, 0.64)	< 0.001
**Social network and Internet browsing**				
	Boys		Girls	
	OR (95% CI)	*P* value	OR (95% CI)	*P* value
Alone	Reference group		Reference group	
Parents only	0.64 (0.57, 0.72)	< 0.001	0.47 (0.43, 0.63)	< 0.001
Friends only	0.17 (0.10, 0.35)	< 0.001	0.42 (0.31, 0.57)	< 0.001
Siblings only	0.94 (0.56, 1.59)	0.84	0.64 (0.43, 0.94)	0.02
Parents & siblings	0.48 (0.42, 0.54)	< 0.001	0.26 (0.19, 0.36)	< 0.001
Other grouping	0.23 (0.13, 0.40)	< 0.001	0.22 (0.14, 0.33)	< 0.001
**Electronic games**				
	Boys		Girls	
	OR (95% CI)	*P* value	OR (95% CI)	*P* value
Alone	Reference group		Reference group	
Parents only	0.59 (0.45, 0.79)	< 0.001	1.17 (0.99, 1.37)	0.05
Friends only	3.23 (2.36, 4.44)	< 0.001	3.12 (1.59, 6.09)	0.001
Siblings only	2.13 (1.43, 3.19)	< 0.001	4.67 (2.78, 7.86)	< 0.001
Parents & siblings	0.41 (0.30, 0.55)	< 0.001	0.95 (0.61, 1.49)	0.84
Other grouping	0.46 (0.30, 0.72)	0.001	0.70 (0.57, 0.86)	< 0.001
**TV-viewing**				
	Boys		Girls	
	OR (95% CI)	*P* value	OR (95% CI)	*P* value
Alone	Reference group		Reference group	
Parents only	4.79 (3.82, 6.01)	< 0.001	4.61 (3.82, 5.57)	< 0.001
Friends only	0.51 (0.33, 0.77)	0.002	0.96 (0.73, 1.27)	0.80
Siblings only	5.43 (3.98, 7.41)	< 0.001	4.59 (3.53, 5.97)	< 0.001
Parents & siblings	4.40 (3.49, 5.57)	< 0.001	5.01 (4.11, 6.10)	< 0.001
Other grouping	1.79 (1.66, 1.93)	< 0.001	1.51 (1.18, 1.94)	0.001

*OR* Odd Ratio, *95% CI* 95% Confidence Interval

On weekend days, compared to the reference category of being alone, all social contexts were associated with lower odds of undertaking any of the behaviours studied in boys and girls, with most of the differences being highly statistically significant. In boys only, being with friends only was associated with higher odds of time spent in phone calls/emails compared to being alone. Being with friends only or siblings only was associated with higher odds of time spent in electronic games in both boys and girls, whilst being with parents or siblings only, parents & siblings and other grouping was associated with higher odds of time spent in TV viewing in boys and girls.

Sensitivity analyses were conducted excluding data collected during August, corresponding to the main school summer holiday in the UK. The overall pattern of findings did not differ meaningfully to our main analysis either for weekdays or weekend days.

## Discussion

This study describes diurnal patterns in adolescents screen behaviours and examines the role of social context in these behaviours separately for week and weekend days. We found screen behaviours peaked in the late afternoon and evening, with TV viewing being most prevalent in both sexes, followed the use of electronic games/apps in boys and social networking sites in girls. Screen activities were mainly reported as being undertaken alone, except for TV-viewing. Being with family members was associated with more time TV-viewing in both sexes on weekdays and weekend days. These strong diurnal and social contextual patterns indicate that behaviour change interventions may be most efficacious if they are targeted at particular times of the day and particular agents, depending on the behaviour of interest.

Television viewing was found to be the main screen activity, rising from the afternoon onwards and peaking in the evening hours for both sexes on weekdays and weekend days. Our findings are in line with a systematic review showing that TV-viewing was the most prevalent behaviour in the hours immediately after school (from 15:00 to dinner time) [12]. This is also consistent with evidence in the field of physical activity which shows that participation in active pursuits declines in the late afternoon and evening [23, 24]. Our findings therefore suggest that adolescents may be substituting active behaviours, for example sports and other non-screen activities with TV viewing in the evenings, and this occurs more frequently as they reach young adulthood. Further, qualitative evidence shows that TV-viewing is a popular family-based activity, mostly used to watch movies in the evenings [25]. Considering that evening screen time may adversely impact sleep [26, 27], our findings suggest that the development of interventions aimed at reducing TV-viewing should be targeted at the evening, although, as discussed below, the impact on family function would require careful consideration.

During the late afternoon and evening on weekdays and the entire waking day at the weekend, the observed increase in time spent TV viewing was accompanied by higher levels of electronic game play in boys and social media use in girls. The differences we observed in electronic gaming and social networking use by sex are consistent with previous studies [28, 29]. Data suggest that electronic game play and social media use occurs throughout the day, though at a relatively low level. This is consistent with survey data showing that 45% of US adolescents are online and open an app on their telephone at least 50 times a day [30]. Further, a systematic review showed that young people spend around 6% of the after-school time in screen behaviours other than TV viewing [12]. Whilst these behaviours might substitute for more physically active pursuits, they are pervasive and become the means for modern youth to connect and communicate with friends online [25], and develop new skills. Interventions to reduce screen time should therefore acknowledge the importance and the role of these screen behaviours in adolescents’ social life, with a goal of the elimination of screen behaviours not therefore being feasible or desirable. Rather there is a need to balance screen time with other activities and support adolescents in establishing a heathy approach to screen use. Understanding co-occurrence or patterns in behavioural transitions would be a valuable adjunct to the data presented in this paper. Sex-specific findings suggest a potential need for tailored interventions for boys and girls by addressing constraints that are unique to, or most pronounced for boys and girls.

Being with family members was associated with more time spent in TV viewing in both sexes on weekdays and weekend days. The scarcity of evidence on the associations of social context with specific screen behaviours makes the direct comparison of our findings with prior research difficult. Nevertheless, other studies have noted that TV-viewing is often a family-based activity, supported by parents as an opportunity for quality family time and communication amongst family members [25, 31]. However, qualitative evidence suggests that TV viewing is often a secondary or background activity alongside mobile phone or tablet use, which may undermine potential benefits associated with family interaction [32]. Considered alongside evidence that having a television in the bedroom, which facilitates viewing alone, is associated with an increased likelihood of being exposed to violent or age-inappropriate content [33], family-based TV viewing may be preferable to that undertaken in other contexts. In a prospective observational study, parental monitoring of children’s media use, encompassing limit-setting and discussion of use/content, was positively associated with a number of social and behavioural outcomes [34]. These findings illustrate the need to work alongside families in the development of interventions to modify children’s screen use, ensuring efforts to limit screen time do not result in unintended adverse consequences on family dynamics or health.

The predominant social context for social network use or internet browsing was alone, whilst making phone calls/sending texts and playing electronic games was more likely to be done in the company of friends and/or siblings, though this varied by sex and day of the week. Numerous studies have reported that social networking and playing video games provide valued opportunities for young people to socialise with friends [35], but it is interesting to observe that this sometimes takes place alone and sometimes in the company of others. Any attempt to modify screen use in this population will need to account for the social function these activities hold in young peoples’ lives. It is also likely that intervention programmes will need to be tailored to the sex- and time-specific (week / weekend) contexts in which these behaviours occur. Qualitative research has shown that young people recognise a range of benefits and problems associated with screen behaviours [32]. Intervention developers should work alongside young people to identify key areas of concern and the most valued outcomes from behaviour change programmes targeting screen behaviours. Our findings indicate that such programmes will need to accommodate the varied social contexts that accompany these behaviours, perhaps drawing upon siblings and friends to support behaviour change.

The study has several strengths and weaknesses. A key strength is the large geographically and demographically diverse sample. In addition, time-use diary data allowed us to study specific screen behaviours and the temporal and social context in which they were undertaken; something which has been little studied in this field to date. Lastly, the reporting of data in screen behaviours separately for weekday and weekend days allowed us to distinguish patterns to better inform the development of interventions. Results should be interpreted with the following limitations in mind. Firstly, data are derived from a British population and, as such, conclusions may not be generalizable to other nations, especially lower income countries with lower adoption of screen behaviours. Secondly, our analytical sample differed in a number of social and demographic characteristics to the wider cohort. Finally, this analysis was not able to account for concurrent screen use, such as using a mobile phone whilst also watching television.

## Conclusion

To our knowledge, this is the first study to use time-use diary data to describe diurnal patterns in adolescents screen behaviours and examine the association of social context with these behaviours. The development of interventions aimed at reducing TV-viewing should be targeted at the evening. Family members and friends may be particularly important targets in behaviour change interventions, but further research is needed to understand the potential impact of interventions to reduce screen time on family functioning and how best to support young people in achieving a healthy balance of screen and non-screen behaviours throughout the day and week.

## Supplementary Information

**Additional file 1: Table 1.** Full list of categories and codes (behaviours) for time-use diary.

## Data Availability

The datasets generated and/or analysed during the current study are available in the UK Data Service repository, [http://doi.org/10.5255/UKDA-SN-8156-7].

## Abbreviations

MCS: Millennium Cohort Study
BMI: Body Mass Index
OECD: Organisation for Economic Co-operation and Development
IOTF: International Obesity Task Force
