# Supplementary material for: The diurnal pattern and social context of screen behaviours in adolescents: a cross-sectional analysis of the Millennium Cohort Study
Source: BMC Public Health. 2022 Jun 7;22:1143. doi: 10.1186/s12889-022-13552-8 (PMC9175381; doi:10.1186/s12889-022-13552-8)
Supplement: Supplementary file 1 — Additional file 1: Table 1. Full list of categories and codes (behaviours) for time-use diary. [file 12889_2022_13552_MOESM1_ESM.docx]

The diurnal pattern and social context of screen behaviours in adolescents: a cross-sectional analysis of the Millennium Cohort Study.

# Additional file 1

Table 1. Full list of categories and codes (behaviours) for time-use diary.

| Categories | Codes |
| --- | --- |
| Sleep and personal care | Sleeping and resting; Personal care |
| School, homework, and education | Homework; In class; School breaks; School clubs; Detention |
| Paid or unpaid work | Paid work; Unpaid work for family or other non-household members |
| Chores, housework, and looking after people or animals | Cooking, cleaning, and shopping for the household; Fixing things around the house, fixing bike, gardening; Looking after siblings in the household; Looking after parent or other adult in the household (medical or personal care); Looking after animals |
| Eating and drinking | Eating or drinking in a restaurant or café; Eating a meal; Eating a snack or having a drink |
| Physical exercise and sports | Cycling; Ball games and training; Jogging, running, walking, hiking; Team ball games and training; Swimming and other water sports; Other physical exercise and other sports |
| Travelling (including walking to school) | Travel by bus, taxi, tube, plane; Travel by car, van (including vehicles owned by friends and family); Travel by physically active means (walk, bike etc.) |
| Social time and family time | Attending live sporting events; Cinema, theatre, performance, gig; Exhibition, museum, library, other cultural events; Shopping; Speaking on the phone; Speaking, socialising face-to-face |
| Internet, TV, and digital media | Answering emails, instant messaging, texting;  Browsing and updating social networking sites (e.g. Twitter, Facebook, BBM, Snapchat);  General internet browsing, programming (not time on social networking sites); Listening to music, radio, iPod, other audio content;  Playing electronic games and Apps; Watching TV, DVDs, downloaded videos |
| Volunteering and religious activities | Volunteering; Religious activities |
| Hobbies and other free time activities | Did nothing, just relaxing, bored, waiting; Hobbies, arts and crafts, musical activities, writing stories, poetry; Reading (not for school) |
| Any other activity | Other activities not listed |
